# Oral Lipid Nanocrystal Amphotericin B (MAT2203) for the Treatment of Invasive Fungal Infections

**DOI:** 10.1093/ofid/ofae346

**Published:** 2024-06-24

**Authors:** Liam M Dalton, Carol A Kauffman, Marisa H Miceli

**Affiliations:** Division of Infectious Diseases, Department of Internal Medicine, University of Michigan Medical School, Ann Arbor, Michigan, USA; Division of Infectious Diseases, Department of Internal Medicine, University of Michigan Medical School, Ann Arbor, Michigan, USA; Division of Infectious Diseases, Department of Internal Medicine, University of Michigan Medical School, Ann Arbor, Michigan, USA

**Keywords:** antifungals pipeline, antifungal treatment, invasive fungal infections, novel antifungal agents, oral amphotericin B

## Abstract

Amphotericin B (AmB) has broad fungicidal activity against many fungi, but the high incidence of adverse events, particularly nephrotoxicity, and the need for intravenous administration restrict its use for many patients. MAT2203, an investigational oral AmB formulation available under a compassionate use program, uses a lipid nanocrystal bilayer structure to deliver AmB with lower toxicity. We present a synopsis of clinical characteristics, treatment course, and outcomes for 5 patients who were treated with MAT2203. Outcomes were positive, with cure of infection noted in 4 patients and improvement in 1 patient who remains on therapy. MAT2203 was well tolerated with only modest gastrointestinal adverse effects. This new oral formulation might provide a safer treatment option for patients requiring extended courses of AmB.

## Teaser

We report on 5 patients treated with MAT2203, an oral amphotericin-B formulation. MAT2203 was well tolerated and effectively treated deep tissue infections due to Fusarium (2) and Rhodotorula (1), Candida krusei urinary tract infection (1) and disseminated histoplasmosis (1).

Amphotericin B (AmB) is a fungicidal agent active against most yeasts and molds. Currently, AmB is available only in intravenous formulations that are often associated with treatment-limiting complications, including renal toxicity and electrolyte abnormalities [1]. MAT2203 (MATINAS Biopharma) is an investigational AmB formulation that uses a rolled phosphatidylserine lipid nanocrystal (LNC) bilayer structure to deliver AmB. This structure allows for oral administration with minimal risk of degradation, as well as very low AmB serum concentrations, resulting in reduced risk of toxicity [2–4]. MAT2203 is currently available under a compassionate use program for treatment of fungal infections in patients in whom other therapies have failed or who have unacceptable toxic effects from currently available treatment options. We present a synopsis of clinical characteristics, treatment course, and outcomes for 5 patients who received MAT2203 administered under this compassionate use program.

## METHODS

Patients were considered for compassionate use MAT2203 if they had a diagnosis of proven/probable invasive fungal disease as defined by European Organization for Research and Treatment of Cancer/the Mycoses Study Group criteria [5] and could not receive standard-of-care antifungal treatment due to lack of response, associated toxic effects, or drug-drug interactions.

Regulatory approval was provided under an emergency investigational new drug application by the US Food and Drug Administration. Local institutional review board approval was obtained for each patient. Patients who met enrollment criteria gave their informed consent for MAT2203 therapy.

## CASE HISTORIES

Findings in the 5 patients are summarized in Table 1.

**Table 1.** Findings in of 5 Patients Treated With MAT2203

Patient	Age/Sex	Organism	Infection Site	Prior Antifungal Therapy (Duration)	MAT2203 Duration	Adverse Effects	Response
1	38/F	*Rhodotorula mucilaginosa*	Bone	L-AmB (4 wk)	24 wk	None	Complete
2	61/M	*Candida krusei*	Bladder	AmB-d (4 d)	2 wk	Moderate diarrhea	Complete
3	40/F	*Fusarium* species	Burn wound	L-AmB (6 d)	17 d	None	Complete
4	48/F	*Fusarium falciforme*	Deep-tissue wound	Voriconazole (4 wk)	25 wk	Nausea, bloating “weird taste”	Complete
5	44/M	*Histoplasma capsulatum*	Disseminated with CNS involvement	L-AmB (6 wk); itraconazole (4 d); L-AmB (13 d)	Ongoing (>28 wk)	None	Improvement; ongoing therapy

Abbreviations: AmB-d, amphotericin B deoxycholate (given intravenously); CNS, central nervous system; F, female; L-AmB, liposomal amphotericin B (given intravenously); M, male.

### Case 1: *Rhodotorula mucilaginosa* Calcaneal Osteomyelitis

**Figure 1.**
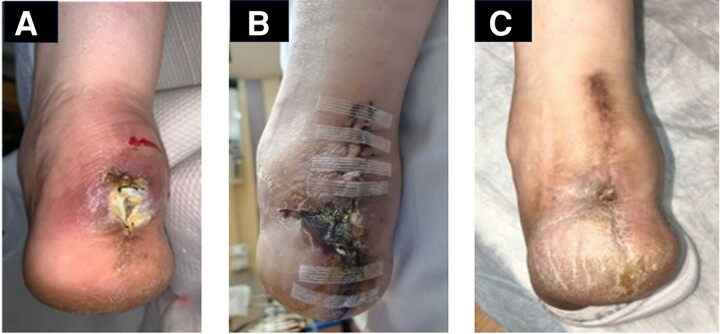
*A*, Chronic nonhealing left calcaneal osteomyelitis just before surgical resection and debridement in patient 1 (19 May 2022). *B,* Minimal improvement after 4 weeks of antimicrobial treatment with meropenem (18 June 2022). *C,* Resolution of calcaneal osteomyelitis after completion of 4 weeks of intermittent intravenous liposomal amphotericin B, followed by 5 months of MAT2203 therapy (20 January 2023).

A chronic purulent nonhealing left calcaneal ulcer developed in a 38-year-old woman with systemic lupus erythematosus (Figure 1*A*). Magnetic resonance (MR) imaging showed calcaneal osteomyelitis with involvement of the Achilles tendon. On 22 May 2022, the patient underwent partial Achilles tendon excision and debridement and partial calcanectomy with vacuum-assisted closure. Intraoperative proximal bone culture yielded coagulase-negative *Staphylococcus* sp, *Campylobacter urealytica*, *Peptostreptococcus anaerobius, and Rhodotorula mucilaginosa*. Therapy with meropenem was begun, but after 4 weeks with no resolution (Figure 1*B*), liposomal AmB (L-AmB) was added (3 mg/kg/dL, given intravenously).

Within 2 weeks after the patient began L-AmB treatment, severe dysgeusia, abdominal pain, and profound hypokalemia and hypomagnesemia developed, requiring insertion of a feeding tube and intravenous electrolyte replacement. Because of her intolerance for L-AmB, which she had received only intermittently over 4 weeks, therapy was initiated with MAT2203 liquid suspension (15 mL [300 mg] 4 times daily). The patient's gastrointestinal symptoms and electrolyte abnormalities had totally resolved within 12 days. By week 12 of MAT2203 therapy, MR imaging demonstrated resolving osteomyelitis, and the surgical site was almost completely healed. The patient was treated with MAT2203 for 24 weeks, with no adverse effects noted (Figure 1*C*). MR imaging repeated 6 months after the conclusion of treatment revealed no signs of osteomyelitis, and when last seen on 2 January 2024 she continued to be well.

### Case 2: *Candida krusei* Hemorrhagic Cystitis

A 61-year-old man with diabetes mellitus type 2, chronic kidney disease (baseline creatinine level before admission, 1.5 mg/dL), and multiple episodes of hemorrhagic cystitis associated with *Candida krusei* infection experienced painful gross hematuria and rising creatinine levels (2.0 mg/dL) on 13 January 2023. Treatment with intravenous micafungin and AmB-deoxycholate bladder irrigation failed to clear his symptoms or eradicate *C krusei.* Computed tomographic urography demonstrated small clots in the bladder, and cultures obtained at cystoscopy yielded *C krusei and* coagulase-negative *Staphylococcus*. Treatment with AmB-deoxycholate was begun (0.6 mg/kg/d, administered intravenously), but after 5 days the patient’s creatinine level, which had decreased to 1.3 mg/dL on 17 January 2023 had risen again to 2.0 mg/dL on 22 January. Because of worsening renal function, treatment with MAT2203 liquid suspension (15 mL [300 mg] 4 times daily for 14 days) was initiated on 24 January 2023. MAT2203 was well tolerated, with the exception of moderate diarrhea. At the end of therapy the patient’s hematuria had resolved, and dysuria and urgency were improved. A urine culture obtained 7 days after initiation of MAT2203 showed no growth, as did follow-up cultures obtained in May, June, and July 2023.

### Case 3: *Fusarium* Species Burn Wound Infection

A 40-year-old woman with paraplegia secondary to a C5–C6 spinal cord injury experienced 34% total body surface area burns on her face, torso, arms, and left leg in a house fire on 5 January 2023. On 22 March 2023 a burn wound infection with a *Fusarium* species developed on the lower part of her left leg. The burn wound was debrided, and oral voriconazole treatment was begun. When the organism was reported as resistant to voriconazole and posaconazole but susceptible to AmB, therapy was changed to L-AmB (5 mg/kg/d, given intravenously). After 6 days, the patient’s creatinine level rose to 1.3 mg/dL (baseline, 0.5 mg/dL), and L-AmB was stopped. Therapy with MAT2203 was started (liquid suspension; 15 mL [300 mg] 4 times daily). While taking MAT-2203, the patient's creatinine level returned to 0.5 mg/dL, and the skin graft site on her left leg was greatly improved; she had no adverse effects related to MAT2203 therapy. After 17 days of MAT2203 therapy, she was transferred to a burn center in her home state to allow continued insurance coverage. MAT2203 was discontinued, and the patient restarted intravenous L-AmB for 2 weeks, with complete resolution of infection. A follow-up report 3 months later revealed that her wound was completely healed.

### Case 4. *Fusarium falciforme* Deep-Tissue Infection

**Figure 2.**
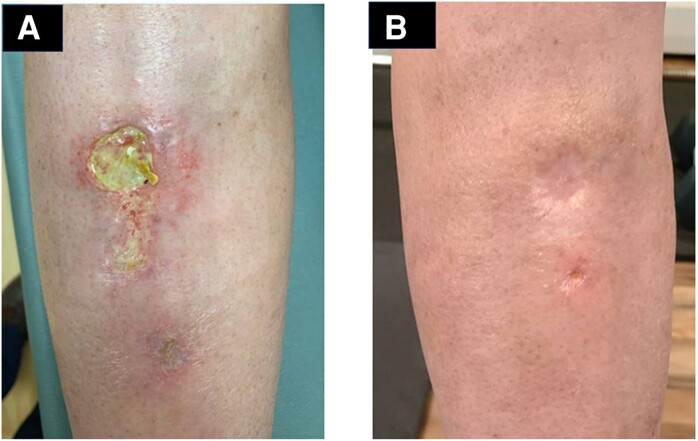
*A*, Chronic ulcerative soft-tissue infection of the right shin in patient 4, a renal transplant recipient showing swelling and purulent drainage 3 months after the initial trauma (24 July 2023). A wound culture yielded *Fusarium falciforme. B,* Soft-tissue infection is completely healed after 23 weeks of treatment with MAT2203 (20 February 2024); MAT2203 was stopped 2 weeks later.

A 48-year-old woman who had received a living-unrelated kidney transplant in 2010 and who was taking everolimus and prednisone, 10 mg daily, injured her right shin on a car door in April 2023. Over the next 3 months, the wound continued to be painful, swollen, and draining purulent material; on 24 July 2023 a wound culture yielded *Fusarium falciforme*, a member of the *Fusarium solani* species complex (Figure 2*A*). Treatment with voriconazole for 2 weeks led to no improvement, and studies showed that the organism was resistant to voriconazole and posaconazole but susceptible to AmB. Because of concern for nephrotoxicity and possible loss of the transplanted kidney, treatment with MAT2203 liquid suspension, 15 mL (300 mg) 4 times daily, was begun on 11 September 2023. While taking MAT2203, the patient noted mild morning nausea, bloating, and a “weird taste” that resolved with gum or candy. The wound gradually improved and was considered to be resolved following 25 weeks of treatment (Figure 2*B*).

### Case 5: Disseminated Histoplasmosis

A 44-year-old man who had received a living-unrelated kidney transplant in 2015 and who was taking tacrolimus, mycophenolate mofetil, and prednisone, 5 mg daily, experienced fatigue and malaise in June 2023. Disseminated histoplasmosis with acute hypoxic respiratory failure and central nervous system involvement was diagnosed. He was treated with L-AmB for 6 weeks. Although he improved clinically, serum and urine *Histoplasma* antigen levels remained ≥20 ng/mL. On 15 August 2023, his therapy was changed to oral itraconazole, 200 mg twice daily.

On 22 August 2023, the patient experienced acute urinary retention with signs of obstructive kidney injury (creatinine 3.8 mg/dL). Lumbosacral MR imaging demonstrated 2 foci of diffusion restriction along the posterior aspect of the thecal sac at L1, with associated nodular enhancement of the cauda equina concerning for leptomeningeal involvement in the context of known central nervous system histoplasmosis. Intravenous L-AmB was restarted, but, given the concern for nephrotoxicity, therapy was changed to MAT2203 liquid suspension, 1(5 mL [300 mg] 4 times daily) on 5 September 2023.

Repeated MR imaging of brain and lumbosacral spine on 1 December 2023 showed resolution of previously noted enhancing lesions in the brain and improvement of cauda equina lesions. The *Histoplasma* serum and urine antigen levels on November 2023 were 1.42 and 5.19 ng/mL, respectively. As of 26 February 2024, the patient was asymptomatic, and he has had no adverse effects from MAT2203. The *Histoplasma* serum and urine antigen levels at the last visit were 0.98 and 2.69 ng/mL, respectively. It is anticipated that the patient will remain on MAT2203 for ≥1 year.

## DISCUSSION

AmB is a broad-spectrum fungicidal antifungal agent that has been used for decades with minimal emergence of resistance and much success in the treatment of a wide variety of serious fungal infections [1]. However, the high incidence of adverse events associated with its use, particularly nephrotoxicity, and the need for intravenous administration have contributed to increasing reliance on azole and 1,3-β-glucan synthase inhibitors in the antifungal armamentarium. Innate or acquired resistance, to azole agents—and less often to 1,3-β-glucan synthase inhibitors—has become increasingly important, and absence of fungicidal activity by these agents to various classes of fungi remains a drawback to their use for certain fungal infections [6–9].

MAT2203 allows AmB to be administered orally, protected from gastric acidity by its insertion within a unique multilamellar spiral crystalline (LNC) nanostructure composed of liposomal preparations of phosphatidylserine stabilized by calcium ions [4]. These LNCs are distributed from the gut into lymphatics and subsequently the bloodstream, where it is hypothesized that they mostly remain intact until taken up by phagocytes and/or infected cells expressing phosphatidylserine on their surface. Once inside the intracellular space with its lower calcium concentration, the LNC structure is disrupted, releasing AmB. Thus, by delivering AmB intracellularly at the site of infection, serum concentrations remain low, decreasing the risk of toxicity [3]. Pharmacokinetic studies have demonstrated MAT2203 to have a large volume of distribution and a particular propensity to distribute to the liver and spleen, further corroborating phagocyte-mediated uptake of this formulation of AmB [3].

Successful treatment of several different fungal infections with oral LNC AmB, including cryptococcal meningitis, invasive candidiasis, pulmonary mucormycosis, and aspergillosis, has been demonstrated in experimental murine models [2, 10–12]. Subsequently, phase I and II trials have been performed in patients with refractory mucocutaneous candidiasis and human immunodeficiency virus (HIV)–associated cryptococcal meningitis [13–15]. Gastrointestinal issues, primarily nausea, bloating, and diarrhea, were the main adverse effects noted in these trials. MAT2203 was associated with lower toxicity and demonstrated survival benefit similar to that of intravenous AmB in patients with HIV-associated cryptococcal meningitis [14, 15]. The phase I trial demonstrated no grade IV or higher adverse events across all dosages, and only a single grade III adverse event occurred [14]. In the phase 2 trial, MAT2203 had significantly lower toxicity at 6 weeks, particularly with regard to hemoglobin (grade III AEs in 21% for MAT2203% vs 43.9% for AmB; *P* = .01) and potassium (5% vs 17%l *P* = .04) [15].

We have noted MAT2203 to be well tolerated with modest gastrointestinal adverse effects. It appears to provide a safer treatment option for patients requiring therapy with AmB for weeks to months, and the oral formulation allows patients to be treated in the outpatient setting. It may be particularly promising for infections that are often resistant to antifungal agents other than AmB, such as those caused by *Fusarium* species.
